# Electroacupuncture for oxaliplatin-induced facial numbness: A CARE-compliant case report

**DOI:** 10.1097/MD.0000000000045454

**Published:** 2025-10-24

**Authors:** Yanbin Ying, Huiting Liu, Qiongying Shen, Yi Liang, Chao Lu

**Affiliations:** a Department of Rehabilitation, The Second Hospital of Jinhua, Jinhua, Zhejiang Province, China; b Zhejiang Chinese Medical University, Hangzhou, Zhejiang Province, China; c Department of Traditional Chinese Medicine, Zhejiang Cancer Hospital, Hangzhou, Zhejiang Province, China.

**Keywords:** case report, chemotherapy, electroacupuncture (EA), facial numbness, oxaliplatin-induced peripheral neuropathy (OIPN)

## Abstract

**Rationale:**

Facial numbness typically arises from disorders affecting the facial or trigeminal nerves, yet chemotherapy-induced facial numbness is exceedingly rare. Herein, we present a CARE-compliant case of a patient with oxaliplatin-induced facial numbness who experienced symptom improvement following electroacupuncture (EA) treatment.

**Patient concerns:**

A 73-year-old patient with rectal cancer developed severe bilateral facial numbness, affecting the periorbital, perinasal, and perioral regions after oxaliplatin-based chemotherapy.

**Diagnoses:**

The patient was diagnosed with facial numbness secondary to oxaliplatin-induced peripheral neuropathy.

**Interventions:**

The patient was treated with EA therapy to improve facial numbness symptoms.

**Outcomes:**

After 3 EA sessions, the patient’s facial numbness improved significantly; a subsequent 3-session course resulted in complete resolution of symptoms.

**Lessons:**

The case suggests that EA may serve as an effective alternative treatment for chemotherapy-induced facial numbness.

## 1. Introduction

Oxaliplatin is a commonly used chemotherapy agent for gastrointestinal malignancies but is associated with significant neurotoxicity.^[1]^ Oxaliplatin-induced peripheral neuropathy (OIPN) occurs in more than 60% of patients receiving oxaliplatin-based anticancer therapy.^[2]^ Though nonfatal, OIPN substantially impairs patients’ health-related quality of life. Classic OIPN manifestations include distal sensory changes in the hands and feet, often described as a “glove-and-stocking” distribution and motor weakness,^[3]^ with less frequent presentations of cranial nerve involvement.^[4]^ Notably, facial numbness, typically attributed to facial or trigeminal nerve pathology, is rarely reported in OIPN. In various publications, acupuncture or electroacupuncture (EA) therapy has been established as a safe and feasible intervention for facial sensory abnormalities in non-chemotherapy settings.^[5,6]^ However, there are few reports on the effect of EA for chemotherapy-induced facial numbness. Here, we described a case of a 73-year-old patient with rectal cancer who developed oxaliplatin-induced facial numbness and achieved complete symptom resolution with EA therapy.

## 2. Case presentation

### 2.1. Patient information

On April 7, 2023, a 73-year-old man presented to the Acupuncture Department of Zhejiang Cancer Hospital with about 1 month of persistent facial numbness attributed to chemotherapy. His medical history was notable for rectal cancer, diagnosed in May 2020, for which he underwent radical resection followed by 6 cycles of the XELOX regimen (oxaliplatin 150 mg plus capecitabine). He then completed regular posttreatment follow-up. In January 2023, follow-up imaging revealed lung metastases from rectal cancer. He underwent thoracoscopic partial lobectomy for metastatic disease at Zhejiang Cancer Hospital in February 2023, with uneventful postoperative recovery. On March 7, 2023, he initiated the first cycle of salvage chemotherapy for recurrent/metastatic disease, again with the XELOX regimen (oxaliplatin 150 mg plus capecitabine), scheduled every 3 weeks.

As early as 2020, the patient reported a history of mild, persistent hand and foot numbness after chemotherapy, which did not interfere with daily activities, so he did not seek any treatment. However, following chemotherapy on March 7, 2023, he developed recurrent hand and foot numbness, along with new-onset facial numbness, predominantly involving the periorbital, perinasal, perioral, and preauricular regions. This was accompanied by mild facial muscle movement restriction with a tightening sensation of facial muscles, which persisted through the chemotherapy interval. The facial numbness severely impaired activities of his daily living, including tooth brushing and eating. After the second cycle of chemotherapy on March 30, 2023, his facial numbness worsened; persistent symptoms also caused insomnia, further disrupting his quality of life. He was referred to the acupuncture department for management of these symptoms.

### 2.2. Diagnostic assessment

On evaluation, the patient reported severe bilateral facial numbness, affecting the periorbital, lateral nasal, preauricular, and perioral regions, persisting throughout the day and significantly impairing his daily activities. Facial nerve function testing demonstrated intact ability to perform voluntary facial movements (eye closure, eyebrow elevation, nose twitching, cheek puffing), though with mild subjective restriction during movement. Tactile and pain sensations were preserved on facial sensory examination. The head computed tomography (CT) was unremarkable. The patient declined facial nerve electromyography. He also reported persistent hand and foot numbness, more severe in the lower extremities. Limb muscle strength and tone were normal on neurological examination. Based on these clinical findings, the patient was diagnosed with facial numbness secondary to OIPN. Using Levi’s Oxaliplatin-Specific Sensory Neurotoxicity Scale (Table 1), his OIPN was graded as 3.

**Table 1:** Levi’s oxaliplatin-specific sensory neurotoxicity scale.

Grades	Severity description
Grade 0	No sensory abnormalities.
Grade 1	Abnormal or dull sensation (caused by exposure to cold), completely resolved within 1 wk.
Grade 2	Abnormal or dull sensation, which can completely subside within 21 d.
Grade 3	Abnormal or dull sensation that cannot completely subside within 21 d.
Grade 4	Abnormal or dull sensation accompanied by functional impairment.

## 3. Intervention and outcome

**Figure 1.**
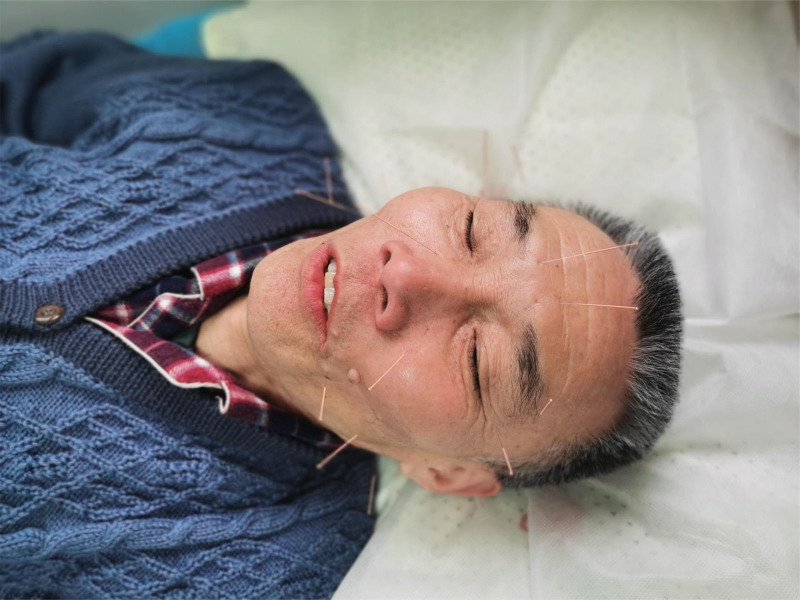
Acupuncture Treatment. The figure depicts the patient undergoing acupuncture therapy.

A professional acupuncturist with 8 years of experience has formulated an acupuncture and EA plan for the patient, with a continuous treatment of 5 days constituting one course of treatment. The acupoints were chosen as Cuanzhu (BL2), Sizhukong (SJ23), Taiyang (EX-HN5), Shangyingxiang (EX-HN8), Quanliao (SI18), Dicang (ST4), and Fengchi (GB20). The location of these acupoints is shown in Table 2 and Figure 1. Disposable acupuncture needles (0.18 mm × 25 mm; Hwato, Suzhou Medical Supplies Factory Co., Ltd., Suzhou, China) were used. The patient was placed in a supine position, and acupoint areas were sterilized. For periorbital acupoints, the patient was instructed to close their eyes during needle insertion. BL2 was needled toward the inner canthus; SJ23 was needled along the temporal side toward EX-HN5; EX-HN8 was needled upward along the bilateral nasal sides. Conventional direct needling was used for all other acupoints. All needles were inserted to a depth of 15 to 22 mm, to make the face of the patient feel an obvious “soreness and distension” sensation (de qi). EA was applied separately to bilateral SI18 and ST4 using an electronic acupuncture device (Hwato SDZ-IIB; Suzhou Medical Supplies Factory Co., Ltd., Suzhou, China) at a frequency of 2 Hz, with intensity adjusted to the patient’s comfort level. Each EA session lasted 30 minutes.

**Table 2:** Location of acupoints.

Acupoints	Location
Cuan Zhu (BL2)	Located in the depression at the medial end of the eyebrow.
Si Zhu Kong (SJ23)	Located in the depression at the lateral end of the eyebrow.
Tai Yang (EX-HN5)	Located between the lateral end of the eyebrow and the outer canthus, about 1 finger-width back.
Shang Ying Xiang (EX-HN8)	Located at the upper edge of the nasolabial groove, on the side of the nostril.
Quan Liao (SI18)	Located on the cheek, in the depression below the zygomatic bone, at the intersection of the vertical line of the outer canthus and the horizontal line of the ala nasi.
Di Cang (ST4)	Located about 0.4 cun lateral to the corner of the mouth, below the zygomatic bone.
Feng Chi (GB20)	Located on the nape, below the occiput, in the depression between the sternocleidomastoid and trapezius muscles.

After 3 consecutive treatment sessions (not completing the full 5-day course), the patient reported complete resolution of facial numbness and restoration of normal daily activities; thus, the patient elected to discontinue treatment. Concurrently, the patient’s hand and foot numbness were addressed via EA, with the treatment protocol referenced from our prior research.^[7]^ After 3 sessions, his hand and foot numbness improved markedly, especially the numbness symptoms in the hands basically disappeared.

**Figure 2.**
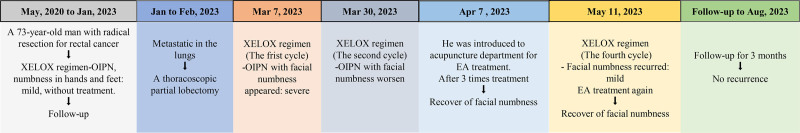
Treatment Timeline. Patient’s disease progression and treatment timeline. OIPN = oxaliplatin-induced peripheral neuropathy.

On May 11, 2023, the patient received the fourth cycle of XELOX chemotherapy. After chemotherapy, his facial numbness recurred but was significantly less severe than prior episodes, with Levi’s Oxaliplatin-Specific Sensory Neurotoxicity Scale rated as grade 1. Three additional sessions of acupuncture/EA per the original scheme resulted in complete resolution of facial numbness (downgraded to grade 0). Throughout the entire treatment process, no adverse events occurred. Subsequent 3-month follow-up (through August 2023) revealed no recurrence of facial numbness (Fig. 2).

## 4. Discussion

Oxaliplatin, a first-line chemotherapeutic agent extensively used in the treatment of colorectal cancer, exerts its anticancer effects primarily by acting as an inhibitor of Deoxyribonucleic Acid (DNA) topoisomerase I. Within cells, oxaliplatin forms stable complexes with DNA and topoisomerase I, disrupting the processes of DNA replication and transcription, thereby effectively inhibiting the proliferation of tumor cells.^[8]^ However, this drug is also associated with a common side effect, OIPN, which, in some cases, can be severe enough to affect a patient’s ability to continue chemotherapy.^[9]^ While the exact pathogenesis of OIPN remains incompletely elucidated, current evidence points to multifactorial contributions.^[10]^ Firstly, oxaliplatin may interfere with the structure and function of neurons by affecting microtubule stability, a crucial component of the cytoskeleton that is essential for maintaining the morphology of nerve cells and transmitting nerve signals.^[8]^ Secondly, the drug may increase oxidative stress by generating excessive reactive oxygen species within cells, which can damage cell membranes, proteins, and DNA, thereby affecting the health and function of nerve cells.^[11]^ Lastly, inflammatory responses also play a role in the development of OIPN, as the drug may trigger the release of pro-inflammatory cytokines from immune cells, which can exacerbate nerve damage and pain.^[12]^ The interaction of these factors may lead to nerve cell damage and dysfunction, resulting in the clinical symptoms of OIPN.

OIPN symptoms predominantly involve the hands and feet, consistent with the classic “glove-and-stocking” distribution, whereas head and facial numbness remains relatively rare. Notably, there are currently no recommended strategies for effective primary prevention of OIPN. In terms of treatment, duloxetine is the only pharmacologic agent with clinical recommendations for OIPN.^[13]^ However, its therapeutic benefits are limited,^[14]^ or bring other adverse effects.^[15]^ Pharmacological treatment of OIPN is rarely limited and has little effect on patients with predominantly numbness rather than pain. This unmet clinical need highlights the importance of exploring novel therapeutic strategies for OIPN. Acupuncture and EA, which have shown safety and feasibility in managing sensory abnormalities, represent promising, viable alternatives worthy of further investigation.

Growing evidence supports the role of acupuncture in addressing chemotherapy-induced peripheral neuropathy (CIPN), including OIPN. Some studies have demonstrated that acupuncture can improve CIPN and is effective in patients with peripheral neuropathy with predominantly numbness in the hands and feet.^[16,17]^ Beyond extremity involvement, acupuncture or EA has also shown distinct advantages in managing facial nerve-related disorders.^[18,19]^ Mechanistically, preclinical research further validates EA’s neuroprotective potential. Electrical stimulation via EA has been shown to modulate microRNA expression, a process that enhances nerve repair.^[20]^ In a rat model of sciatic nerve injury, EA significantly increased the number of myelinated fibers, axonal and fiber diameters, and myelin sheath thickness, to improve nerve injury-related dysfunction.^[21]^ These findings provide a biological basis for EA’s efficacy in OIPN, where nerve damage and demyelination are thought to contribute to sensory symptoms.

Previous clinical studies by our team also demonstrated that EA was a potential therapy for treating CIPN, with low-frequency (2 Hz) EA exhibiting more significant efficacy in improving chemotherapy-induced sensory nerve symptoms,^[22,23]^ and these findings were later validated in the treatment of Utidelon-induced peripheral neuropathy.^[24]^ This evidence directly informed the selection of 2 Hz EA in the current case, aligning with our established low-frequency EA practice for sensory-predominant CIPN. In addition, our team has also reported on EA’s efficacy in chemotherapy-induced cranial nerve dysfunction. For example, we previously documented a breast cancer patient who developed oculomotor nerve paralysis after paclitaxel chemotherapy and achieved complete recovery with EA treatment.^[25]^ This is consistent with another study showing satisfactory outcomes of EA for chemotherapy-induced abductor nerve paralysis.^[5]^ For this patient, his oxaliplatin-induced facial numbness was presumably attributed to chemotherapy-induced trigeminal or facial nerve dysfunction, pathophysiologically analogous to the aforementioned cranial nerve palsies. The complete resolution of symptoms after EA treatment in this patient further extends this body of evidence, highlighting EA’s potential in addressing a broader spectrum of chemotherapy-induced peripheral nerve disorders.

## 5. Conclusion

This case suggests that acupuncture may have a potential role in treating chemotherapy-induced facial numbness. As it is only a single case, no definitive conclusions can yet be drawn, and further randomized controlled studies as well as systematic evaluations are needed to provide a high-quality basis.

## Acknowledgments

We are grateful to all the researchers, including the physicians, and technicians, who participated in this study.

## Author contributions

**Funding acquisition:** Yi Liang, Chao Lu.

**Investigation:** Huiting Liu.

**Methodology:** Chao Lu.

**Resources:** Chao Lu.

**Validation:** Yi Liang.

**Writing – original draft:** Yanbin Ying, Qiongying Shen, Chao Lu.

**Writing – review & editing:** Yanbin Ying, Huiting Liu, Chao Lu.
